# Supplementary material for: Housing-temperature reveals energy intake counter-balances energy expenditure in normal-weight, but not diet-induced obese, male mice
Source: Commun Biol. 2022 Sep 10;5:946. doi: 10.1038/s42003-022-03895-8 (PMC9464191; doi:10.1038/s42003-022-03895-8)
Supplement: Supplementary file 2 — Supplementary Information [file 42003_2022_3895_MOESM2_ESM.pdf]

Supplementary Information

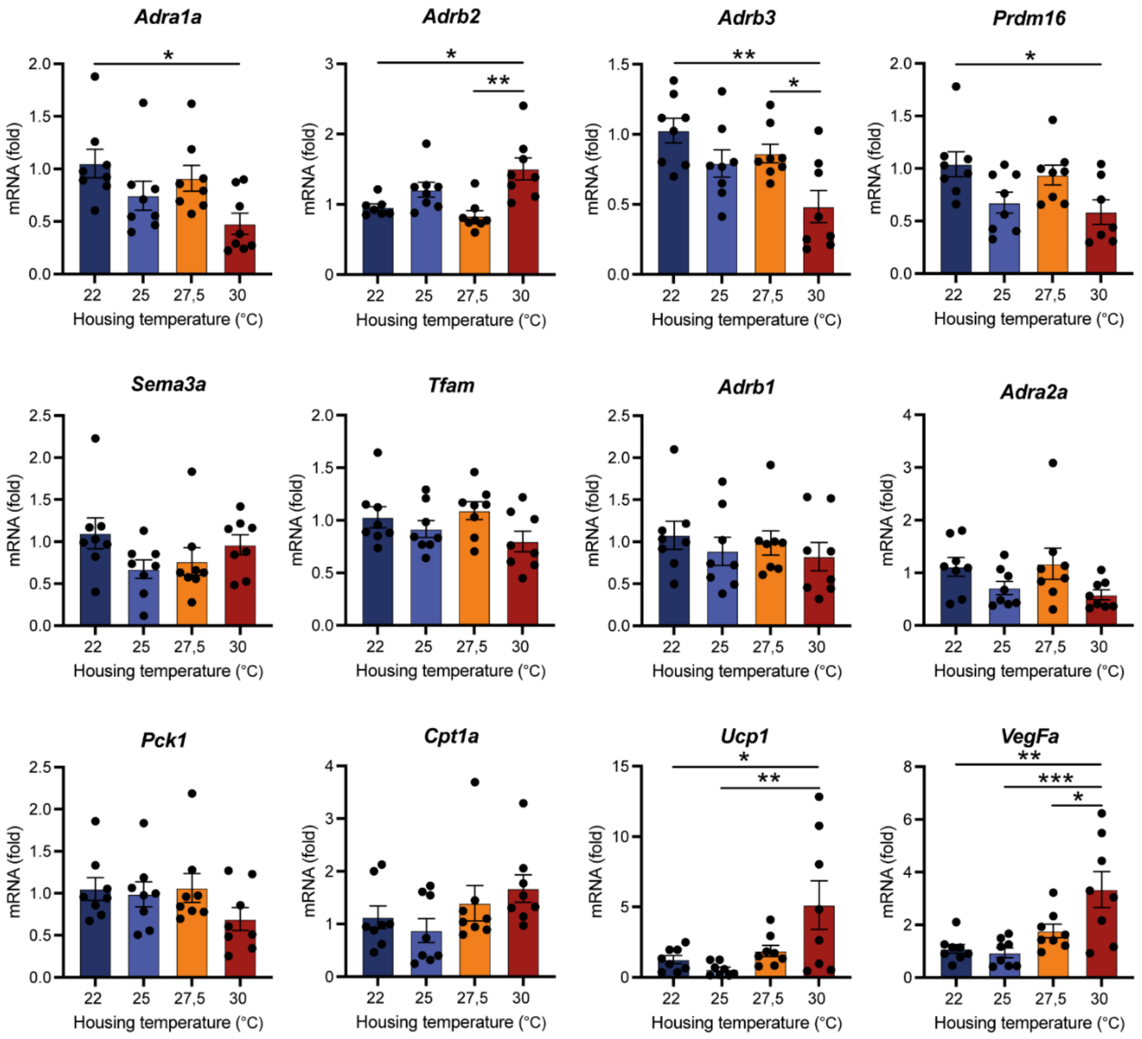

Supplementary figure 1: Expression of key genes regulating BAT activity/function. Data are expressed as means±SEM relative to average expression level in 22°C group. Statistical significance was tested by Oneway ANOVA followed by Tukey multiple comparison test when appropriate. Dots indicate different animals. Abbreviations: *Adra*: Adrenoceptor Alpha 1A, *Adrb*: Adrenoceptor Beta, *Prdm15:* PR domain containing 16, *Sema3a*: Semaphorin-3A, *Tfam:* Transcription Factor A, Mitochondrial), *Pck1*: Phosphoenolpyruvate Carboxykinase 1, *Cpt1a:* Carnitine palmitoyltransferase 1a, *Ucp1*: Uncoupling protein 1, *VegFa*: Vascular Endothelial Growth Factor A.

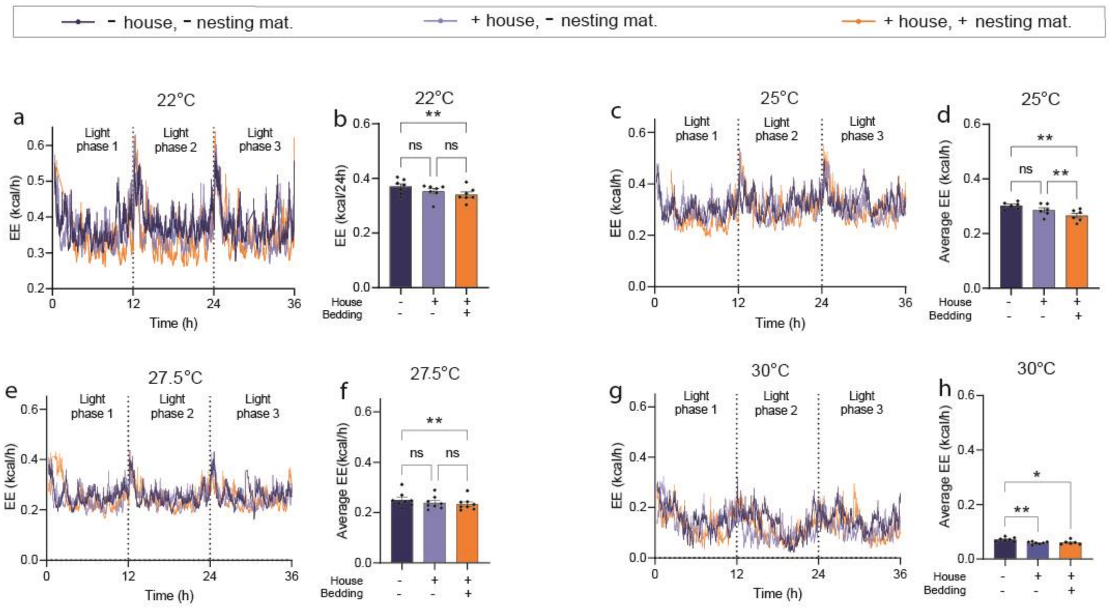

Supplementary figure 2: Importance of houses and nesting material for expenditure in mid-old lean mice during light phase. Energy expenditure data (kcal/h) are shown at 22, 25, 27.5 and 30°C under conditions without house and nesting material (dark blue), with house but without nesting material (light blue) and with house and with nesting material (orange). Data are a representation of the data in figure 4, but in this case focusing on the light phase only (0-12, 24-36, 60-72h in fig. 4). Data are presented as means±SEM. Dots in bar graphs indicates individual mice. Average EE (kcal/h) values were calculated over the entire experimental period, covering three consecutive light phases as indicated above. **P<0.01, ***P<0.001, n=7-8.

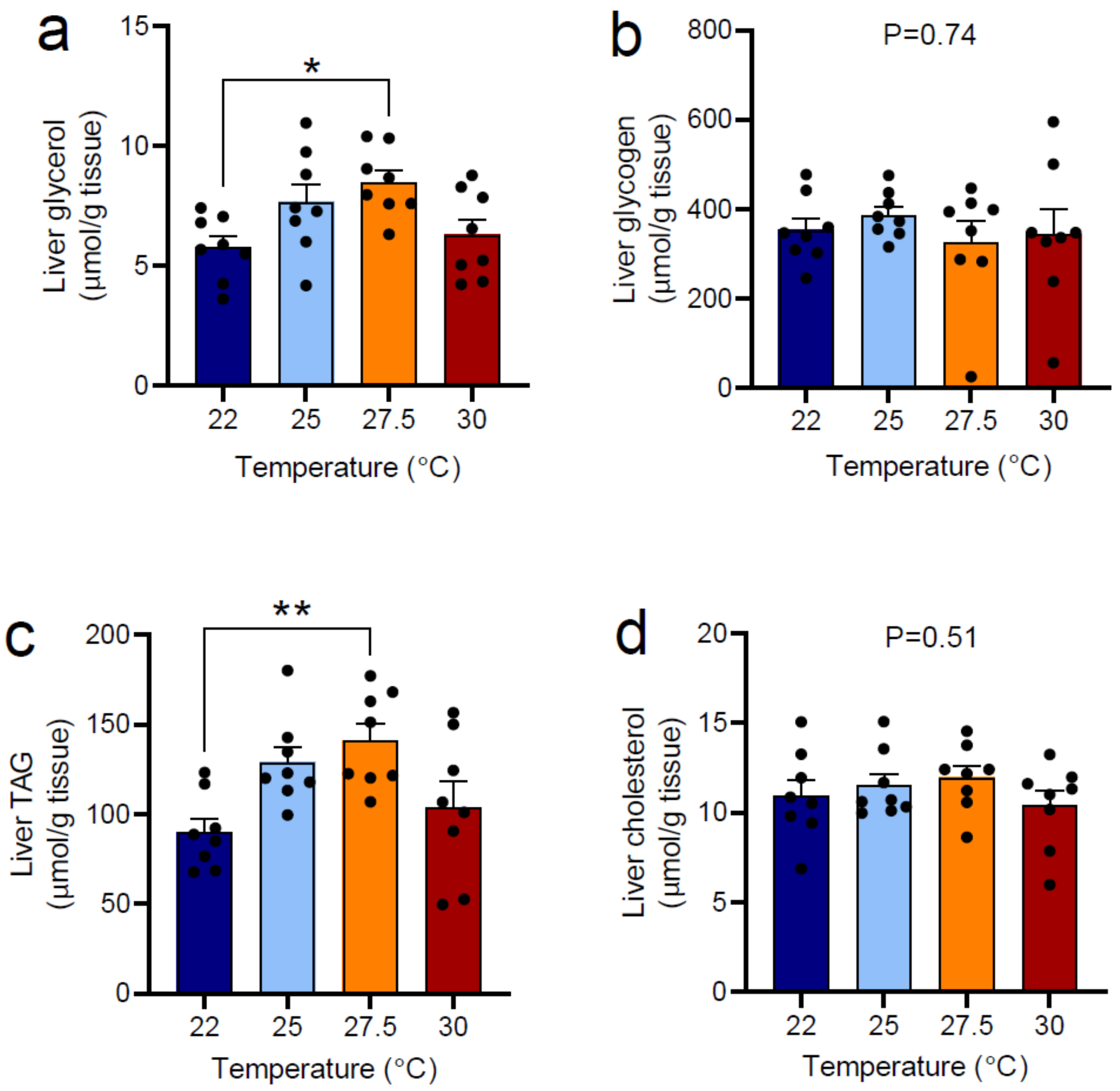

Supplementary figure 3: Effects of housing temperature on extractable concentrations of liver glycerol, glycogen, TAG and cholesterol. Data are shown as means+SEM. Dots in bar graphs indicates individual mice. *P<0.05, **P<0.01, n=7-8.

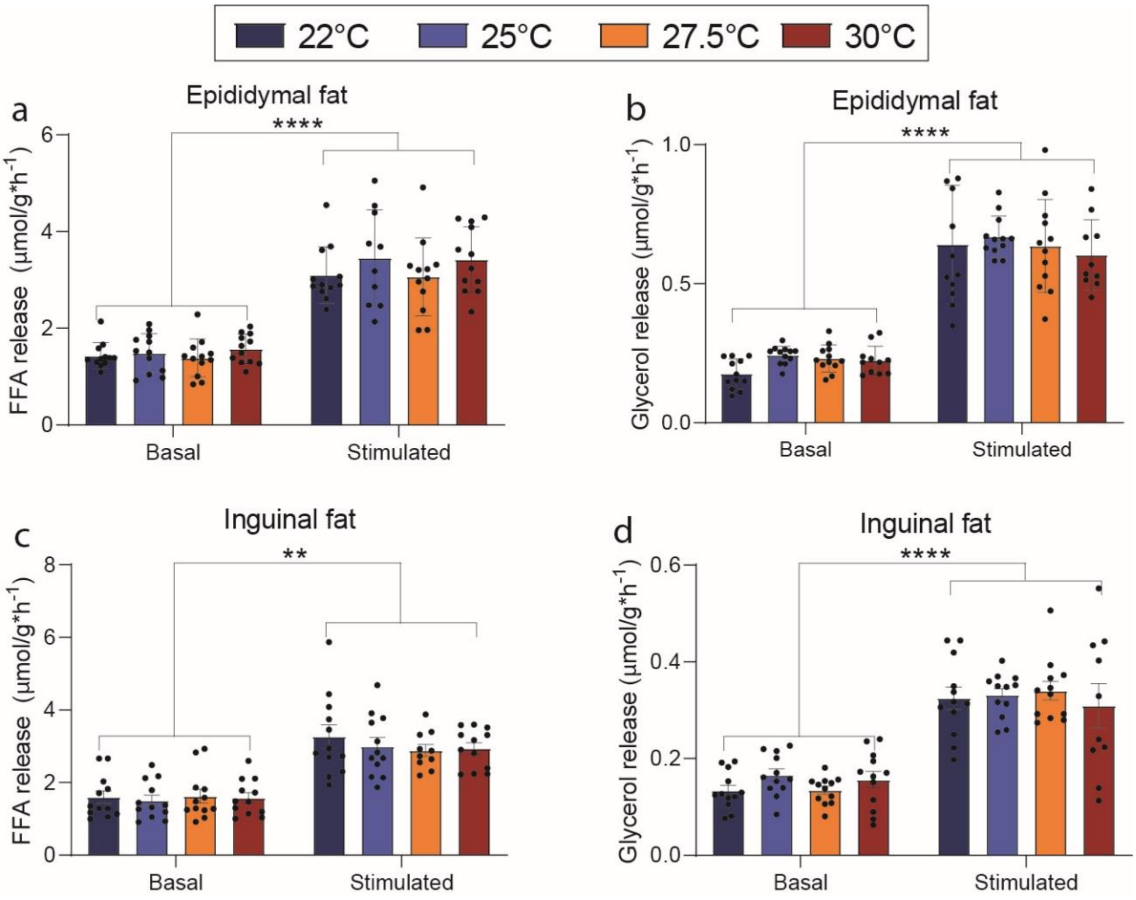

Supplementary figure 4: Increasing housing temperature does not result in altered ex-vivo lipolyisis from epidymal and inguinal fat isolated from diet-induced obese mice. Free-fatty-acid (FFA) release and glycerol release (both µmol/g*h^{-1}) from epididumal fat (A,B) and inguinal fat (C,D) are shown at baseline and in response to stimulation with 1 µM isoprenaline. Fat was isolated from the same diet-induced obese mice used for the studies in fig. 1, 3, 5, and 6. Data are expressed as means±SEM. **P<0.01, ****P<0.0001, n= 12 replicates obtained from 4 animals.

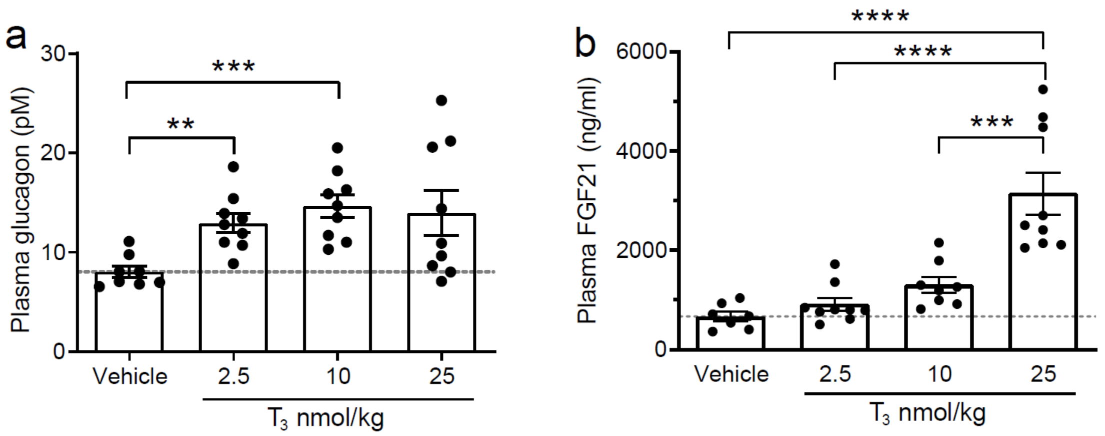

Supplementary figure 5: Effects of Triiodothyrodine (T_3) administration on plasma glucagon and FGF21 concentrations. Diet-induced obese (DIO) male mice (~40 g, n=9), housed at room temperature and with ad libitum access to 45% high-fat-diet, were treated once daily for four weeks with subcutaneous injection of T_3 at following doses: 2.5, 10, or 25 nmol/kg. Plasma was obtained from non-fasted anesthetized mice by retroorbital vein puncture. Mice were anesthetized with isoflurane. Plasma was collected into EDTA-coated tubes, which were immediately put on ice and was centrifuged within half an hour. T_3 was formulated in following buffer: 0.05% (w/v) Tween-80, 70 mM NaCl, 50 mM Phosphate, pH=8.15. Plasma FGF21 and glucagon concentrations were quantified as described in material and methods in the main text. A: Plasma glucagon (pM), B: Plasma FGF21 (ng/ml). **P<0.01, ***P<0.001, ****P<0.0001. Test for statistical significance was done by One-way ANOVA followed by Tukey multiple comparison test.

Supplementary Methods

Importance of ambient temperature for glucose tolerance in normal-weight and DIO male mice:

Besides regulating EE, ambient temperature may also regulate other aspects of metabolism, including post-prandial glucose metabolism, possibly through regulation of the secretion of glucometabolic hormones. To test this hypothesis, we finalized the temperature study in both normal-weight and DIO mice by challenging them with an oral glucose load (2 g/kg body weight, 50% (w/v) glucose, 4 ml/kg). At this stage, mice had been housed at least 8 days at the respective temperaturesMice were fasted for 6-7h (food was removed at start of light cycle (0600h)) and were placed in their cages in a procedure room (22°C) two hours before the challenge to reduce the influence of acute responses to changes in ambient temperature. Blood was collected from the tail vein into 5µl heparinized glass capillary tubes and immediately suspended in hemolysing buffer (250µl System Solution EKF Diagnostics, Germany) and analysed using glucose oxidase method (Biosen S-line Glucose Analyser, EKF Diagnostics, Germany).

Importance of ambient temperature for lipids and hormones in plasma and lipids in the liver under short morning-fast conditions:

Plasma used for these analyses was terminal bleeding samples obtained the day after the OGTT described in section 2.6. Mice had their food removed by start of light cycle (0600h). Collection of blood and organs were started 3h after and lasted ~2h. Order of takedown was done in a systematic manner to ensure that fasting time did not differ between groups. Plasma samples were, therefore, from 3-5h fasted mice. To minimize acute effects driven by changes in ambient temperature, mice were left in their cages in the SABLE system at their respective housing temperatures until takedown and maximum in the procedure room (22°C) for 10 min. At take down, mice were anesthetized by isoflurane, blood was collected into pre-chilled EDTA-coated tubes, which was instantly put on ice and centrifuged (6000 x g, 4°C, 5 min) within 15 min. Plasma was transferred into fresh eppendorf tubes or tubes with added sodium fluoride (final concentration in whole blood: 5 mg/ml). Plasma was stored at -20°C until analysis. Plasma concentrations

of free-fatty acids (FFA), glycerol, HDL, triglycerides (TG), cholesterol, 3-hydroxybuturate (3-HB), alanine aminotransferase (ALT), and aspartate aminotransferase (AST) were quantified using a Cobas 6000 analyzer (F. Hoffmann-La Roche AG, Basel, Switzerland), following instructions provided by the manufacturer. FFA and glycerol were quantified on the Cobas 6000 analyzer in samples that were supplemented with sodium fluoride for stabilization. Liver concentrations of extractable glycerol, glycogen, TG and cholesterol was measured by same procedure and was normalized to tissue weight (µmol/g wet tissue). Free glucose concentrations was subtracted from reported glycogen concentrations. Endogenous concentrations of mosue insulin, C-peptide, glucagon and leptin was measured using a custom developed Mesoscale (MSD) multiplex assay which is a multiarray assay with electrochemiluminescence readout. Mouse FGF21 was quantified using a commercial kit: from Biovendor (Mokrá Hora, Czechia, Cat. no. RD291108200R). Lower limit of quantification was: insulin: 3.6 pM, C-peptide: 6.7 pM, glucagon: 1.4 pM, leptin: 1.8 pM, and FGF21: 20 pg/ml.2.8

Ex-vivo lipolysis assay:

Fragments of inguinal and epididymal adipose tissue were excised immediately after euthanizing the mice and placed in HBSS supplemented with 0.1% FFA-free bovine serum albumin (Sigma) and 10 mM Hepes (Life Technologies) at room temperature. Tissue was cut into ~100 mg explants, washed in the buffer mentioned above and incubated in 100 µl of HBSS supplemented with 2% albumin (assay buffer) for 1 hour to generate basal lipolysis samples. After that, the tissue was placed in fresh assay buffer (100 µl) containing 1 µM isoproterenol and incubated at 37º for 30 minutes to start the stimulated lipolysis. Hereafter, the buffer was replaced with fresh stimulation buffer and incubated for 1 hour. Glycerol and FFA content were quantified as described in section 2.7 and were normalized to tissue weight (g). Plates were shaken on an orbital shaker (low speed 150 rpm) for 20 s after medium addition and before the collection.

Expression of thermogenic genes in brown adipose tissue:

Brown adipose tissue (BAT) harvested on the day of study termination was immediately transferred to eppendorf tubes containing RNAlater and stored at -20°C until analysis. RNA was extracted using RNeasy Mini Kit (Cat. No. 74106, Qiagen, Hilden, Germany)

and cDNA was synthesized using iScript™ Reverse Transcription Supermix (Cat. No. 1708840, Bio-Rad Laboratories, inc. CA, USA) following manufacturer's instructions. Expression of 12 key genes regulating thermogenic BAT activity was determined by quantitative real-time PCR on ViiA-7, using 1 ng of cDNA mixed with TaqMan™ OpenArray™ Real-Time PCR Master Mix (Applied Biosystems™, Thermo Fisher Scientific, USA) and TaqMan™ probes listed in supplementary table 1. Expression levels of BAT genes are expressed as relative expression levels using ΔΔCt-method, using *Ywhaz and Hprt* as housekeeping genes for all target genes with an exception of *Ucp1* where *18s* was used. 22°C-housed group was used as control for normalization of mRNA folds.
